# The role of neutrophil extracellular traps in antiphospholipid syndrome

**DOI:** 10.3389/fimmu.2026.1800055

**Published:** 2026-05-12

**Authors:** Chunyao Ren, Youge Su, Hongbin Li, Tingting Ren

**Affiliations:** 1 Department of Rheumatology and Immunology, The Affiliated Hospital of Inner Mongolia Medical University, Hohhot, China; 2 Inner Mongolia Autonomous Region Key Laboratory of Pathogenesis and Immunodiagnosis of Rheumatic and Immune Diseases, Key Laboratory of Autoimmune and Rheumatic Diseases of Inner Mongolia Medical University, Hohhot, China

**Keywords:** antiphospholipid syndrome, neutrophil, neutrophil extracellular traps, pathological pregnancy, thrombotic events

## Abstract

Antiphospholipid syndrome (APS) is an acquired systemic autoimmune disorder characterized clinically by recurrent arterial and venous thrombosis and/or adverse pregnancy outcomes, and laboratory-defined by persistently positive antiphospholipid antibodies (aPLs). Neutrophils are the most abundant white blood cells in the circulation. In APS patients, aPLs abnormally activate neutrophils, causing them to transition from a quiescent state to a highly activated state, which is a central mechanism in disease pathogenesis. Neutrophil extracellular traps (NETs) are reticular structures composed of DNA, histones, and granular proteins released by neutrophils under stress conditions. As a crucial immune mechanism for neutrophil function, NETs play a vital role in anti-infection and immune responses. However, when overactivated or lacking inhibition, NETs can mediate the development of various diseases. Recent studies indicate that neutrophils and NETs occupy a central position in APS pathogenesis, promoting thrombosis and contributing to pathological processes such as placental dysfunction. This review summarizes the role of NETs in the development and progression of APS.

## Introduction

APS is an acquired systemic autoimmune disorder characterized by recurrent arterial/venous thrombosis and/or pregnancy morbidity, along with persistent laboratory evidence of antiphospholipid antibodies (aPLs) (1). Common laboratory tests include lupus anticoagulant (LA), anti-β2-glycoprotein I antibody (anti-β2GPI), and anticardiolipin antibody (aCL) (2). According to the clinical manifestations, APS can be classified into thrombotic APS (TAPS), obstetric APS (OAPS), and catastrophic APS (CAPS). As one of the common acquired thrombophilias, TAPS can involve arteries, veins, and microvessels, with thrombosis characterized by recurrence and occurrence at multiple sites (3). OAPS manifests as unexplained fetal death after 10 weeks of gestation, premature delivery due to eclampsia or severe preeclampsia before 34 weeks, or three or more consecutive spontaneous abortions in early pregnancy (2). A minority of patients develop CAPS, which involves microvascular thrombosis in three or more organs within a short period, often triggered by infection, with a mortality rate as high as 50% (4, 5). In addition to the above manifestations, APS can also be accompanied by non-criteria symptoms such as thrombocytopenia, livedo reticularis, cardiac valve lesions, aPL-associated nephropathy, cognitive dysfunction, and chorea (6).

The pathogenesis of APS has not been fully elucidated. Studies demonstrate that injecting mice with affinity-purified anti-β2GPI from APS patients directly enhances thrombosis (7–9). This intervention exhibits a more pronounced pro-thrombotic potential compared to administration of aCL or antiprothrombin antibodies (7). These antibodies orchestrate a proinflammatory and procoagulant phenotype in platelets, endothelial cells, and monocytes by recruiting and activating tissue factor (TF), which is expressed by neutrophils, thereby driving immunothrombosis (10–12). Complement activation occupies a central position in pathogenesis, as its inhibition protects against both thrombosis and pregnancy complications in animal models, a finding corroborated by human studies linking complement deposition to thrombotic risk (13–15). APLs induce vascular endothelial dysfunction, leading to trophoblast apoptosis and obstruction of placental blood flow (16). Furthermore, genetic associations with HLA class II and non-HLA loci like STAT4 implicate adaptive and innate immune dysregulation in APS susceptibility, though large-scale genetic studies are still needed (17, 18).

Upon stimulation, neutrophils can undergo a unique form of cell death called NETosis, releasing web-like structures composed of decondensed chromatin decorated with cytotoxic and prothrombotic proteins (19). A growing body of evidence indicates that aPLs can directly or indirectly (e.g., via complement activation) induce NETosis (20). A consistent finding across multiple studies is an elevated NETs burden in APS patients. This is evidenced by increased levels of various circulating NETs biomarkers—such as cell-free DNA (cfDNA), myeloperoxidase-DNA (MPO-DNA) complexes, and citrullinated histone H3 (CitH3)—in patient plasma, as well as upregulated expression of NETs-related genes (e.g., PAD4, MPO mRNA) in circulating neutrophils (21–24). Importantly, the elevation of certain markers correlates with clinical severity. For instance, patients with recurrent thrombosis exhibit significantly higher plasma levels of MPO-DNA complexes and higher MPO mRNA expression in circulating neutrophils than those with a single thrombotic event (22). Furthermore, *in vitro* and animal model studies provide mechanistic support: anti-β2GPI/β2GPI complexes can directly induce NETs formation in healthy human neutrophils (25), and the administration of aPLs or anti-β2GPI antibodies in mice promotes NETosis and thrombus formation, which can be mitigated by NETs inhibitors like DNase I (21). Neutrophils and the NETs they release have emerged as a central axis in APS pathogenesis (26, 27), exhibiting a primed and hyperactive phenotype (28).

Our review systematically summarizes the critical role of NETs in both thrombotic and obstetric APS, detailing the mechanisms by which aPLs activate neutrophils, induce NETosis, and how the released NETs drive thromboinflammation and placental injury. Furthermore, it highlights NETs and their components as promising therapeutic targets beyond conventional anticoagulation.

## Mechanisms of NETs formation in APS

### Activation of neutrophils in APS

Neutrophils, the most abundant circulating leukocytes, are classic effector cells of the innate immune system. Research into the role of neutrophils in APS dates to the 1990s. A pivotal study by Arvieux et al. (29) demonstrated that murine monoclonal antibodies (mAbs) against human β2GPI, which possess lupus anticoagulant activity, could activate human neutrophils in a concentration-dependent manner upon binding to β2GPI. This activation, manifesting as degranulation, increased hydrogen peroxide production, and elevated cytosolic Ca^2+^, was shown to depend on the engagement of Fc gamma receptors (FcγR), specifically FcγRII, on neutrophils. Studies indicated that APS patient serum could trigger complement activation, modulating neutrophils to adopt a procoagulant phenotype, thereby inducing TF expression and forming a vicious cycle of inflammation and coagulation (13, 30). The role of complement identified in animal models has also been validated in APS patients (31, 32). Additionally, neutrophils form aggregates with platelets via P-selectin/P-selectin glycoprotein ligand-1 (PSGL-1) interactions, releasing various cytokines and chemokines such as platelet factor 4 (PF4) and high mobility group box 1 (HMGB1). These factors can further activate neutrophils, establishing a positive feedback loop that amplifies local inflammation and thrombosis (33, 34). Knight et al. (35) conducted a systematic transcriptomic study on neutrophils from primary APS patients, revealing a pro-inflammatory gene expression signature in neutrophils characterized by widespread upregulation of genes related to interferon signaling, defense response, and cell adhesion. López-Pedrera et al. (36) examined the expression of 45 splicing machinery components in immune cells from 45 APS patients and found that downregulation of components such as SKIP-SNW1 (a splicing factor involved in transcriptional regulation), SND1 (staphylococcal nuclease domain-containing protein 1, involved in RNA processing), and CUGBP (CUG-binding protein, an RNA-binding protein regulating mRNA stability) in neutrophils was associated with increased thrombosis risk. Furthermore, hypomethylation of developmental pluripotency-associated protein 3 (a regulator of DNA methylation in germ cells) and protein tyrosine phosphatase non-receptor type 2 (a negative regulator of cytokine signaling that modulates immune responses) connected with thrombosis and inflammation, have been identified in APS neutrophils (37). Collectively, these findings delineate a multifaceted landscape of neutrophil activation in APS, spanning receptor-mediated signaling, inflammatory amplification loops, and molecular reprogramming that together orchestrate the heightened propensity for NETosis and thrombotic complications.

### Production of NETs in APS

In the context of APS, neutrophils exhibit an abnormally hyperactive state (28). The process by which neutrophils produce NETs, accompanied by cell lytic death, is called NETosis, a cell death program distinct from apoptosis and necrosis (38). This process primarily occurs via two activation pathways. The first, a slow lytic pathway (typically requiring 2–4 hours), is dependent on the nicotinamide adenine dinucleotide phosphate (NADPH) oxidase complex. It is triggered by diverse stimuli, including pathogens, phorbol esters, cholesterol crystals, autoantibodies, or immune complexes, which induce reactive oxygen species (ROS) generation through NADPH oxidase activation. The released ROS activates the downstream receptor-interacting protein kinase 3-mixed lineage kinase domain-like pseudokinase (RIPK3-MLKL) cascade, while also acting on neutrophil elastase (NE) and myeloperoxidase (MPO), increasing nuclear membrane permeability and promoting chromatin decondensation. ROS further activates peptidylarginine deiminase 4 (PAD4), an enzyme that catalyzes the conversion of arginine to citrulline on histone H3. This citrullination reduces the positive charge of histones, weakening their interaction with DNA and further facilitating chromatin decondensation. Ultimately, nuclear and granular membranes disintegrate, leading to the extracellular release of a DNA-protein web-like structure and resulting in neutrophil death (39). The second, a rapid vital pathway, is NADPH oxidase–independent. In this route, neutrophils activated through Toll-like receptor (TLR) or complement receptor can form NETs without requiring the NADPH oxidase complex (28). For instance, ionomycin, a bacterial ionophore, induces a calcium influx. The increased intracellular calcium binds to and activates PAD4, directly driving histone citrullination and chromatin decondensation. In this pathway, NETs are extruded without immediate plasma membrane rupture, allowing neutrophils to remain viable and retain migratory and phagocytic functions temporarily (40).

## NETs and TAPS

Current research suggests that the mechanisms of thrombosis in APS involve two main aspects: on one hand, aPLs interact with vascular endothelial cells, neutrophils, monocytes, platelets, and complement, forming a complex network through the release of cytokines, chemokines, etc., continuously amplifying inflammatory and coagulation responses (30, 41). On the other hand, the “second-hit” hypothesis posits that in the presence of aPLs, a “second-hit” such as infection, surgery, trauma, or pregnancy, can activate endothelial cells, platelets, monocytes and neutrophils etc. Against the background of a hypercoagulable state induced by aPLs, excessive activation of the coagulation system triggered by these multiple cell types disrupts the balance between coagulation and anticoagulation, ultimately leading to thrombosis (17, 42, 43).

In thrombotic APS, NET formation is significantly increased and promotes thrombosis through multiple pathways. NETs can promote thrombosis by simultaneously activating the extrinsic and intrinsic coagulation pathways. In the extrinsic pathway, NETs enrich TF and promote its expression. Additionally, NE on their surface can cleave tissue factor pathway inhibitor (TFPI), further enhancing TF activity (44). TF binds to coagulation factor VII (FVII) and then triggers activation of the coagulation system, promoting thrombosis (45). Furthermore, complement components on the surface of neutrophils interact with TF, activating the complement cascade, leading to the generation of C5a (13). C5a activates neutrophils via its receptor C5aR, inducing TF expression, increasing ROS production, triggering inflammation, and injury (46). APL-induced NETs, by carrying TF and TF-positive microparticles, significantly shortened plasma clotting time (25). The expression of TF on NETs has been documented in APS patients, and the causal role of NETs in activating the extrinsic pathway *in vivo* has been primarily established in murine models of APS, with supporting mechanistic data derived from non-APS settings.

Regarding the intrinsic pathway, DNA and RNA from NETs can serve as surfaces for contact activation, promoting the conversion of factor XII (FXII) to its active form, FXIIa, which then activates FXI and FIX, thereby amplifying the coagulation cascade (46). Additionally, histones and NE on NETs can directly activate platelets and FV and FVIII. NE and proteinase 3 (PR3) can cleave ATIII and TFPI, thereby inhibiting physiological anticoagulant mechanisms (47). Meanwhile, MPO promotes fibrin formation and thrombus stability by oxidatively modifying fibrinogen (48). One study has shown that FXIIa levels are elevated in APS patients and correlate positively with citrullinated histone H3 (CitH3) and MPO-DNA complexes, which are well-established markers of NETs (49). However, direct evidence that NETs drive FXII-dependent thrombosis specifically in APS remains largely extrapolated from studies in other thrombotic disorders.

Histone H3 on NETs can also bind and neutralize tPA, thereby inhibiting plasmin generation (50). NE can cleave fibrinogen and plasminogen, generating antifibrinolytic substances that further hinder plasmin generation and activity, inhibiting the fibrinolytic process (51). NETs can promote the release of plasminogen activator inhibitor-1 (PAI-1), inhibiting fibrinolytic activity (52). One study shows that histone-DNA complexes in APS patients are negatively correlated with fibrinolytic activity, which means NETs accumulation in APS patients leads to a significant reduction in fibrinolytic activity, increasing the risk of thrombosis (50). Using DNase I to degrade the DNA backbone of NETs was able to restore fibrinolysis inhibited by aPLs, suggesting a key role for NETs in APS fibrinolytic dysfunction (53). These findings explain the characteristics of APS thrombi: their difficulty in dissolving spontaneously and their propensity to recur. The inhibitory effects of NETs on fibrinolysis have been demonstrated using plasma from APS patients and purified components (24); however, the relative contributions of histones, NE, and other NETs-associated molecules to the fibrinolytic defect in APS patients warrant further investigation.

NETs are instrumental in propagating the endothelial dysfunction and prothrombotic diathesis central to APS. Their constituent histones and proteases inflict direct damage on endothelial cells, compromising vascular integrity and increasing permeability (54). One study found that aPL-induced NETs can downregulate endothelial cell expression of thrombomodulin (TM) and endothelial protein C receptor (EPCR), inhibiting the protein C anticoagulant system (55). Furthermore, NETs promote endothelial cell expression of von Willebrand factor (vWF) and P-selectin, enhancing platelet adhesion and activation (56). Yang et al. (57)confirmed that NETs activate endothelial cell pyroptosis via the TLR4/ROS/NLRP3 pathway, releasing TF-rich microparticles. Therefore, NETs-mediated endothelial dysfunction is an important pathological link promoting the occurrence and development of thrombosis in APS.

HMGB1, interleukin-1α (IL-1α), IL-33, etc., on NETs can activate endothelial cells and immune cells, promoting the release of pro-inflammatory cytokines. These inflammatory mediators can further stimulate neutrophils to release NETs, forming a positive feedback loop (58). Additionally, NETs amplify inflammatory responses by activating C3 and C5. Complement system activation can further promote the activation and chemotaxis of inflammatory cells, exacerbating local inflammation. The interaction between inflammation and coagulation is an important characteristic of APS thrombosis (59). By carrying inflammatory mediators and procoagulant substances, NETs become the core of “immunothrombosis”. This explains why anticoagulation therapy alone is sometimes insufficient to completely prevent APS thrombosis recurrence, and anti-inflammatory therapy may provide additional benefit (60).

Platelet activation by aPLs represents a critical step in the pathogenesis of thrombotic APS, as activated platelets subsequently engage neutrophils through multiple receptor–ligand pairs. Among these, the CD40-CD40L axis serves as a key mediator. Engagement of CD40L on platelets with CD40 on neutrophils promotes platelet–neutrophil aggregation and triggers autophagic responses within neutrophils. This autophagy is essential for the subsequent release of NETs enriched with biologically active TF, which in turn drive thrombin generation and propagate thromboinflammation. Notably, disruption of CD40-CD40L interactions—using a specific inhibitor *in vitro* or an anti-CD40L antibody *in vivo*—markedly reduces platelet–neutrophil aggregates, suppresses autophagy, blocks NETs release, and diminishes NETs-induced thrombin generation (61).

In addition to these receptor-mediated mechanisms, genetic factors further modulate NETs formation. Petrus et al. (62) demonstrated that the NCF1–339 polymorphism, which reduces NADPH oxidase-derived ROS production, is closely associated with heightened interferon activity and altered NETs formation in patients with systemic lupus erythematosus (SLE). Notably, this ROS-deficient genotype is significantly linked to the presence of antiphospholipid antibodies and secondary APS, suggesting that NCF1–339 may represent a key genetic determinant bridging the shared pathological pathways of SLE and APS, including autoantibody production, impaired clearance of apoptotic cells, and aberrant NETs formation. Collectively, these findings underscore that NETs lie at the nexus of platelet–neutrophil crosstalk and thromboinflammation in APS. Both receptor-dependent activation cascades and genetic predispositions converge to promote NETs release, thereby establishing a prothrombotic environment that perpetuates vascular injury and thrombotic risk.

In APS, the degradation of NETs is inhibited. Leffler et al. (63) observed that the ability of APS patient serum to degrade NETs was significantly lower than that of healthy controls, and the degree of NETs degradation was weakly negatively correlated with anti-NETs IgG antibody levels. Patients who were anti-dsDNA positive tended to have higher levels of anti-NETs antibodies. Zuo et al. (64) also confirmed impaired NETs degradation in APS patients, finding that this phenomenon was closely associated with the presence of anti-NETs IgG antibodies. Further experiments showed that depletion of anti-NETs IgG antibodies partially restored serum NETs degradation ability, suggesting that these antibodies may have a stabilizing effect on NETs, protecting them from degradation. It is noteworthy that Leffler’s study distinguished between primary and secondary APS, revealing an association in both groups that was more pronounced in secondary APS (typically associated with SLE). Furthermore, Zuo’s work suggested that the implicated antibodies likely target shared antigenic epitopes (e.g., nucleic acid/histone complexes) rather than APS-unique mechanisms, underscoring potential immunopathological links between APS and other systemic autoimmune diseases.

**Figure 1:**
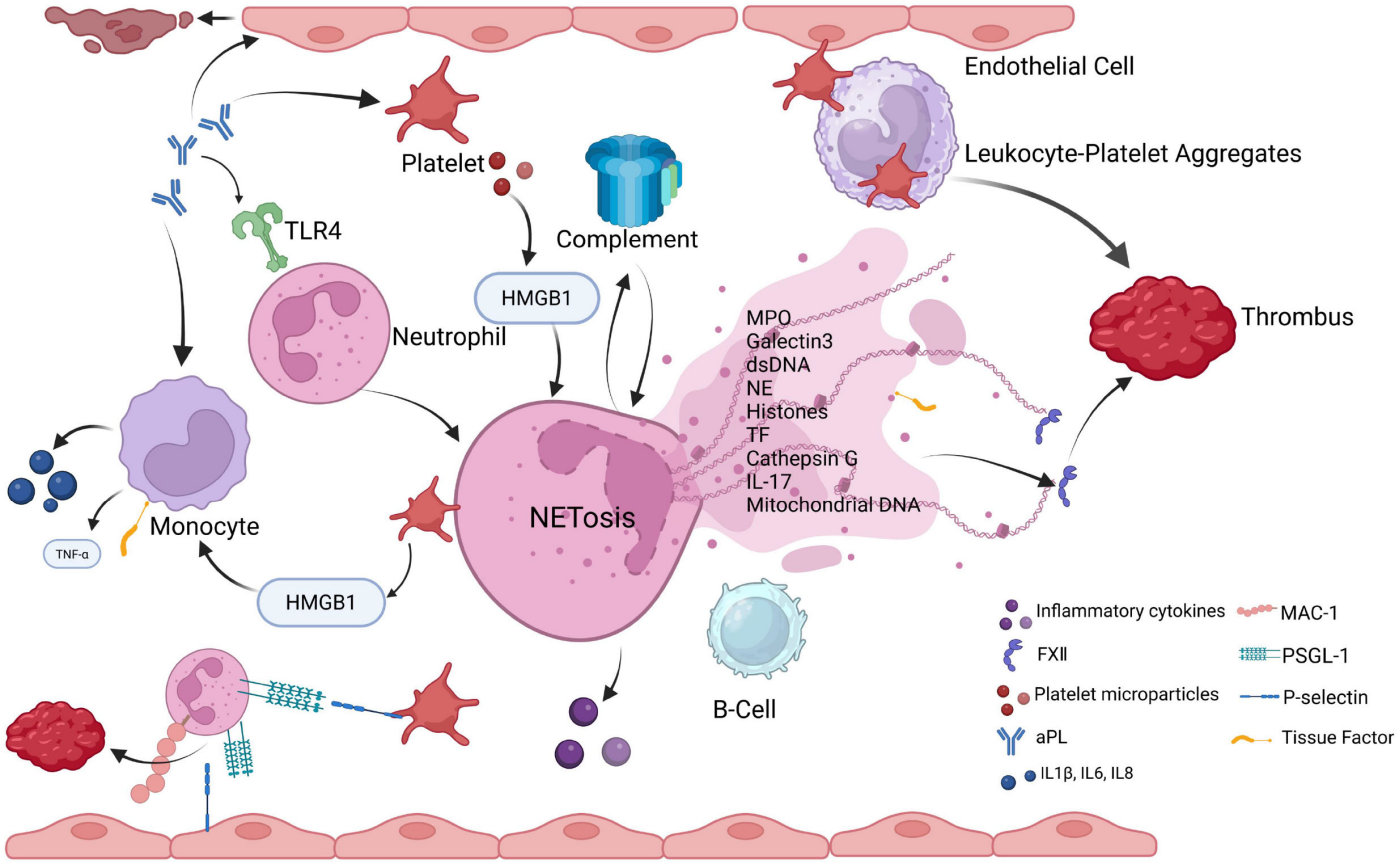
This schematic depicts the central role of NETs in driving thrombosis in APS. APLs activate neutrophils via TLR4, triggering ROS burst and NETosis, which releases chromatin, histones, NE, MPO, TF, and mitochondrial DNA. Activated platelets bind neutrophils via P-selectin/PSGL-1 and MAC-1 to form aggregates, releasing HMGB1 that further amplifies monocyte TF expression and NETosis. NETs components co-stimulate B cells via TLR9/TLR4, while complement activation and exposed TF enhance procoagulant activity, driving a synergistic inflammation-thrombosis cascade that disrupts hemostasis and promotes thrombus formation. Created in BioRender. chubyao, R. (2026). https://BioRender.com/d0dt4ee.

In summary, these studies provide convergent evidence that NETs are elevated in APS, correlate with thrombosis, and contribute to thrombo-occlusive events. However, most of the current evidence has been derived from animal models, and the major limitations of these models must be acknowledged. First, most animal studies use passive antibody transfer, which may not fully recapitulate the chronic, polyclonal autoimmune response seen in patients. Second, correlations from cross-sectional human studies do not establish causality. Third, while anti-NETs antibodies are found to impair NETs clearance, their definitive role as pathogenic drivers versus secondary epiphenomena remains unresolved. Finally, the relative contributions of DNase I dysfunction, anti-NETs antibodies, and other serum factors to impaired NETs degradation in APS are not yet fully characterized. **Figure 1** is a diagram illustrating the mechanisms linking NETs to thrombotic APS.

## NETs and OAPS

OAPS is defined as the association of persistent aPLs with specific pregnancy morbidity, representing a major cause of acquired thrombophilia in pregnancy. Historically, the classification of OAPS has evolved. Initial criteria, the 2006 Sydney revised classification, defined obstetric morbidity primarily as three or more consecutive spontaneous miscarriages before 10 weeks, one or more unexplained fetal deaths beyond 10 weeks, or a premature birth before 34 weeks due to severe preeclampsia or placental insufficiency (65). This definition was later expanded to recognize a broader “non-criteria” spectrum, including early preeclampsia, placental abruption, and other complications, even in women without prior thrombosis (2). A significant advancement came with the 2023 ACR/EULAR Antiphospholipid Syndrome Classification Criteria, which introduced a weighted, points-based system for risk stratification. In this framework, different clinical manifestations contribute variably to the diagnostic score. Pregnancy morbidity features are assigned specific weights: early recurrent miscarriage (1 point), unexplained fetal death at or beyond 10 weeks (2 points), and premature birth before 34 weeks of gestation due to severe preeclampsia or placental insufficiency, which carries a high weight (4 points) in the current classification criteria, underscoring its recognized severity and strong association with the underlying pathogenic mechanisms of APS (2). This weighted approach facilitates a more precise and standardized diagnosis, integrating clinical severity into the classification process. Recent research finds that APS may also be associated with other pregnancy complications, such as HELLP syndrome (hemolysis, elevated liver enzymes, and low platelets), placental abruption, intrahepatic cholestasis of pregnancy, and oligohydramnios etc. although these manifestations have not yet been incorporated into formal diagnostic criteria (66). Currently, relatively clear mechanisms include five main aspects: imbalance in the maternal-fetal interface immune microenvironment (67, 68), trophoblast cell dysfunction (69), vascular endothelial injury (70), genetic susceptibility (71), and gut microbiota dysbiosis (72). Beyond these core pathways, the disease has also been linked to broader systemic disturbances, including endocrine imbalances, infectious triggers, and abnormal amino acid metabolism (40).

The maternal-fetal interface is formed by direct interactions between maternally derived decidual stromal cells and immune cells, and embryo-derived trophoblast cells. This interface constitutes a dynamic immune microenvironment, composed of immune cells and cytokines, which plays essential roles in pregnancy establishment, maintenance, and the initiation of labor, thereby functioning as a critical placental barrier (40). Neutrophils exhibit distinct functional profiles across different stages of pregnancy. During early pregnancy, they contribute to immune tolerance and tissue remodeling, whereas in late pregnancy, they are involved in processes such as cervical ripening and uterine contraction through the release of cytokines and chemokines, thereby supporting normal physiological parturition (73). When the maternal-fetal interface immune system is overactivated, it leads to massive NETs release. The NETs formation process generates large amounts of ROS, leading to intracellular metabolic disturbances, organelle dysfunction, and ultimately injury to various cells in the maternal-fetal interface microenvironment, resulting in pregnancy-related diseases, including preeclampsia and recurrent miscarriage. Compared to healthy pregnant women, OAPS patients exhibit a lower threshold for NETs secretion and a significant increase in NETs within the placental intervillous space, thereby triggering local excessive inflammatory responses, damaging vascular endothelial cells, and impairing trophoblast invasion and migration, which ultimately contribute to pathological pregnancy outcomes (67).

ROS induces intracellular metabolic disturbances and organelle dysfunction, ultimately leading to damage to diverse cell types within the maternal–fetal interface microenvironment and contributing to pregnancy-related disorders such as preeclampsia and recurrent miscarriage (74). NETs disrupt pregnancy maintenance through multiple interrelated mechanisms. First, the histones and proteases they carry damage trophoblasts and endothelial cells directly and activate the complement system to produce C5a, thereby exacerbating local inflammation (75). Second, components such as TF in NETs can activate the coagulation cascade, promoting placental microvascular thrombosis and obstructing blood flow in the placental intervillous space, thereby directly affecting fetal blood supply (76). Additionally, NETs themselves can physically entrap and exacerbate placental tissue damage, triggering interstitial inflammation and trophoblast apoptosis (77). Collectively, these pathological processes lead to placental dysfunction, characterized by impaired spiral artery remodeling and reduced placental perfusion, ultimately resulting in clinical complications including recurrent fetal loss, severe preeclampsia, fetal growth restriction, and placental abruption (78, 79).

Preterm birth (PTB), defined as delivery before 37 completed weeks of gestation, constitutes a major cause of neonatal morbidity and mortality worldwide (17, 80). The primary etiological pathways encompass spontaneous preterm labor and preterm premature rupture of membranes (PPROM). In this context, clinical data indicate that peripheral blood neutrophil counts and the neutrophil-to-lymphocyte ratio are significantly elevated in PTB and PPROM patients, suggesting a key role for neutrophils in this pathological process (81). Pathological evidence of chorioamnionitis exists in over 40% of spontaneous preterm birth cases, indicating that this inflammatory condition, characterized by neutrophil infiltration and activation, may be the underlying mechanism for PPROM and subsequent PTB (13). Overactivated neutrophils not only release pro-inflammatory factors like IL-1β, IL-6, IL-8, and TNF-α but also directly participate in tissue damage through the release of NETs (82). Additionally, bacterial components like lipopolysaccharide (LPS) can stimulate fetal membrane tissue and induce neutrophils to release NETs via an early non-lytic pathway (82). The local accumulation of these NETs is implicated in membrane rupture, thereby contributing to PTB (82). In patients with OAPS, the incidence of PTB is markedly elevated, with studies reporting rates ranging from 14.5% to 48%—significantly higher than in the general obstetric population (83, 84). A systematic review and meta-analysis further indicated that women with APS have a 1.89-fold increased risk of preterm delivery (85).

**Figure 2:**
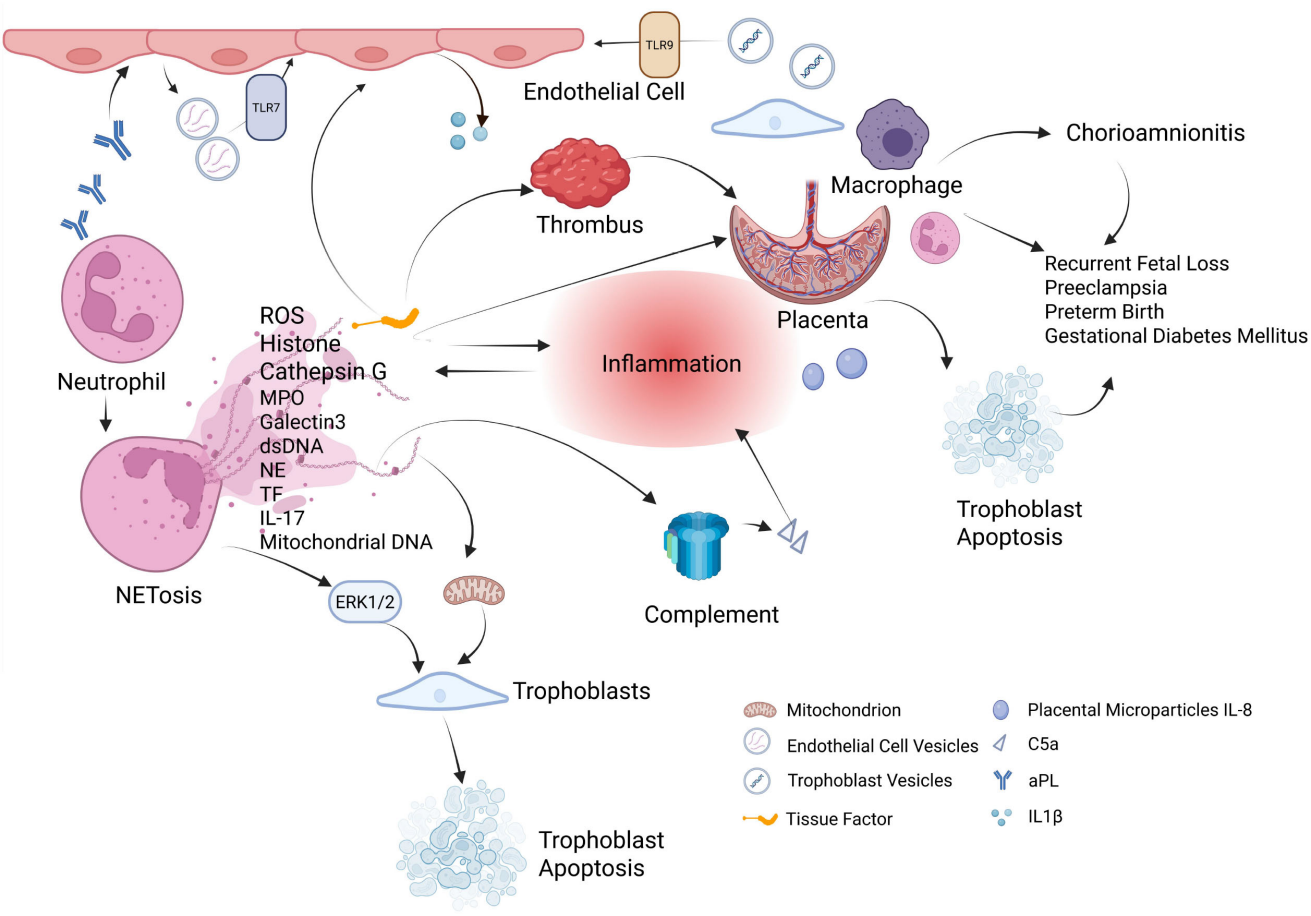
This schematic depicts how NETs mediate obstetric APS. APLs activate neutrophils via TLRs, triggering ROS burst and NETosis, releasing chromatin, histones, NE, MPO, TF, and mitochondrial DNA. NETs components damage trophoblasts and endothelial cells, while TF initiates coagulation and complement activation generates C5a to amplify inflammation. NETs also induce trophoblast apoptosis via mitochondrial and ERK1/2 pathways, impairing placental function. Placental microparticles and IL-8 further promote NETs formation, and aPL-induced extracellular vesicles activate endothelial cells via TLR7/TLR9 to intensify inflammation. These processes collectively drive chorioamnionitis, placental dysfunction, and prothrombotic states, leading to recurrent fetal loss, preeclampsia, preterm birth, and gestational diabetes mellitus. Created in BioRender. chubyao, R. (2026). https://BioRender.com/m2unay9.

As introduced earlier, preeclampsia, especially early-onset preeclampsia, is a common and severe manifestation of OAPS (86, 87). Placental microparticles and IL-8 from PE patients can activate neutrophils, inducing NETs formation. The presence of NETs in the placental interstitium may lead to hypoxia-reperfusion injury, placental infarction, fibrin deposition, and systemic endothelial cell injury, thereby triggering or exacerbating hypertension (77). Furthermore, fetal and maternal cf-DNA levels are significantly elevated in PE patients, suggesting placental injury and NETs formation (88). One study found that non-lytic NETosis, present in normal pregnancy, is elevated in preeclampsia and is associated with an increased risk of thrombosis and pregnancy complications (89). A major limitation is that existing studies do not clarify whether NETs drive these complications through APS-specific pathogenic mechanisms or instead represent a common terminal pathway of placental dysfunction. **Figure 2** illustrates the mechanism diagram of NETs in obstetric APS.

## NETs and CAPS

CAPS is a rare manifestation of APS. Its core lies in the aPL-triggered explosive thrombotic-inflammatory cascade, leading to widespread small vessel emboli throughout the body, ultimately rapidly progressing to multi-organ failure (90, 91). The incidence of CAPS is about 1%, and the prognosis is extremely poor, with mortality as high as 33% in international cohort studies, rising to 48% if secondary to SLE (90). Typical clinical manifestations of CAPS are caused by multi-organ microvascular thrombosis, commonly including acute renal failure, pulmonary embolism, alveolar hemorrhage, stroke, seizures, myocardial pathology, and thrombocytopenia, usually triggered by infection, autoimmune disease, or other procoagulant stressors (92, 93). In the CAPS disease process, pathological features often resemble those of acute thrombotic microangiopathy (TMA). One study shows that plasma levels of NETs markers are significantly elevated in TMA patients, and NETs, through proteases like NE, among their components, degrade endogenous anticoagulants like thrombomodulin, and activate FXII via their DNA backbone, exacerbating the “thrombotic storm” in CAPS (94). Furthermore, studies found that plasma DNase1 activity, responsible for degrading NETs, is significantly impaired in some TMA patients. Reduced plasma DNase1 activity may lead to ineffective clearance of NETs, allowing them to persist in circulation, promoting microvascular thrombosis in TMA patients (92, 95). The extremely low incidence of CAPS precludes the conduct of relevant cohort studies. Moreover, existing case reports have not included assessments of NETosis, and therefore, no further data are currently available.

## Progress in treatments targeting NETs

Approximately 30% of patients with APS do not respond to existing anticoagulant therapy, underscoring the need for new treatment options (96). Standard anticoagulation uses vitamin K antagonists (e.g., warfarin) or low molecular weight heparin, requiring INR monitoring to balance thromboprophylaxis and bleeding risk. Although direct oral anticoagulants (DOACs) offer advantages like ease of use and no need for routine monitoring, studies show they may increase arterial thrombotic risk in high-risk APS patients (especially those triple antibody positive or with arterial thrombotic history) (97). 2019 EULAR Adult Antiphospholipid Syndrome Management Guidelines recommend vitamin K antagonists as the first choice for high-risk patients, while DOACs are only considered for low-risk patients without a history of arterial events (98, 99).

In recent years, the relationship between APS and NETs has gradually gained attention. Increasing evidence indicates that NETs participate in APS pathogenesis and suggests that inhibiting NETs may be a potential therapeutic strategy (100). A study by Bertolaccini et al. (101)demonstrated that hydroxychloroquine (HCQ), in addition to its immunomodulatory effects, prevents thrombosis by inhibiting complement activation, which is a key driver of neutrophil recruitment and activation. By reducing complement-derived signals such as C5a, HCQ mitigates neutrophil stimulation, thereby indirectly suppressing NETosis, reducing TF expression, and attenuating inflammatory pathways critical to APS pathogenesis. This mechanism is essential for preventing both thrombotic events and pregnancy-related complications in APS, including fetal loss and placental abnormalities (102). In obstetric APS, HCQ demonstrates therapeutic benefits. When combined with anticoagulant therapy, HCQ contributes to improved pregnancy outcomes. A study by Jamilya et al. (103) reported that among 217 aPL-positive women who received HCQ in addition to standard therapy with low-molecular-weight heparin (LMWH) and low-dose aspirin (LDA), 182 (83.8%) ultimately achieved live births. Specifically, in a subset of 41 women with prior pregnancy failures on anticoagulant therapy alone, the addition of HCQ resulted in 32 live births (78%). The combination of HCQ with LMWH and LDA also improved obstetric outcomes in another 32 women, contributing to the overall high live birth rate. These findings suggest that the combination of LMWH, LDA, and HCQ represents a promising therapeutic strategy for refractory obstetric manifestations associated with aPL. In CAPS, treatment strategies have expanded from traditional anticoagulation to multi-target interventions, including the anti-CD20 monoclonal antibody rituximab and complement inhibitors. Among these, the complement C5 inhibitor eculizumab, which blocks the core step of complement activation, offers a new direction for refractory CAPS (104, 105). Statins, besides lipid-lowering, exert anti-inflammatory and endothelial function-improving effects by inhibiting the mTOR pathway, providing a theoretical basis for adjuvant therapy in APS (106). These drugs targeting immune and inflammatory pathways, together with anticoagulation, constitute a new comprehensive management landscape for APS.

PAD4 is a key enzyme in the NETs formation process, and inhibiting PAD4 can reduce the release of NETs (107). Studies have shown that the PAD4 inhibitor Cl-amidine effectively reduces NETs formation and attenuates arterial thrombosis in a FeCl3-induced arterial thrombosis model (108). Similarly, GSK484 has been shown to decrease NETs release, lower inflammatory cytokine levels, and protect myocardial tissue in a myocardial infarction model (109). In addition, studies have found that NETosis and thrombosis are significantly reduced in PAD4-deficient mice. Specifically, 48 hours after inferior vena cava stenosis, the thrombus formation rate was less than 10% in PAD4-deficient mice, compared with 90% in wild-type mice. Moreover, extracellular citrullinated histone H3 was detectable only in thrombi from wild-type mice, confirming that PAD4-mediated chromatin decondensation plays a critical role in venous thrombosis (107).

Targeting downstream steps of NETsosis, ROS inhibitors (like N-acetylcysteine) can effectively block NETs formation by scavenging free radicals, potentially offering a new therapeutic approach for APS (17, 110). Scholars have found that defibrotide, an adenosine receptor agonist, was shown to inhibit NETosis and venous thrombosis in an APS model by agonizing the adenosine A2A receptor (28). This is strongly supported by the work of Ali et al., who demonstrated that a specific adenosine A2A receptor agonist (CGS-21680) potently inhibited NETosis and thrombus formation in mice injected with APS patient IgG, providing a direct pharmacological proof-of-concept for this approach (111). In the study by Carlos Pérez et al. (112), healthy monocytes were pretreated with coenzyme Q10 prior to exposure to IgG from APS patients. The findings demonstrated that oxidative perturbations in leukocytes from APS patients are closely associated with a pro-inflammatory and pro-atherothrombotic state, and that coenzyme Q10 treatment could effectively prevent or reverse these abnormalities. Natural compounds with antioxidant properties are also under investigation. For instance, taxifolin, a flavonoid, was recently shown to inhibit NETosis by activating the Nrf2 antioxidant pathway. It conferred protection in both lupus and APS mouse models, reducing thrombotic manifestations, positioning it as a promising candidate for further therapeutic development (113).Meng H. et al. (114) established a murine model of deep vein thrombosis via inferior vena cava stenosis and administered IgG derived from patients with APS. In mice treated with APS-IgG, the thrombus formation rate exceeded 90%, however, administration of DNase I immediately after surgery significantly reduced the thrombus formation rate to approximately 33% within six hours (114).

However, current therapeutic strategies targeting NETs degradation in APS face many challenges, such as the stability of DNase I, administration routes, etc. Moreover, given the multifactorial nature of NETs-driven thromboinflammation, combining DNase I with complementary agents—such as PAD4 inhibitors or ROS scavengers—may offer synergistic effects and represent a more effective strategy for clinical translation. **Table 1** and **Table 2** provides a summary of therapeutic agents targeting NETs.

**Table 1:** Summary of drugs targeting NETs for thrombus treatment in APS.

Experimental type	Drug	Mechanism of action	Experimental model	Application status
Cell experiment	N-acetylcysteine (NAC)	Scavenges ROS and blocks the downstream process of NETs formation	*In vitro* neutrophil culture	Preclinical study
	CGS-21680 (adenosine A2A receptor agonist)	Activates the adenosine A2A receptor pathway and inhibits NETosis	*In vitro* neutrophils stimulated with APS-IgG	Preclinical study
	Taxifolin	Activates the nuclear factor erythroid 2-related factor 2 (Nrf2) antioxidant pathway and inhibits NETosis	*In vitro* neutrophils combined with lupus/APS model cells	Preclinical study
Animal experiment	PAD4 inhibitors (Cl-amidine, GSK484)	Inhibit PAD4 enzyme activity and reduce NETs release	PAD4-knockout mice, APS thrombosis model	Effective in animal experiments; not entered clinical stage
	N-acetylcysteine (NAC)	Scavenges ROS and blocks NETs formation	Mouse APS thrombosis model	Effective in animal experiments
	Defibrotide	Activates the adenosine A2A receptor pathway, and inhibits NETosis and venous thrombosis	Mouse APS model	Effective in animal experiments
	CGS-21680	Activates adenosine A2A receptor, and inhibits NETosis and thrombosis formation	Mouse model injected with APS-IgG	Effective in animal experiments
	Taxifolin	Activates the Nrf2 pathway and inhibits NETosis	Lupus/APS mouse model	Effective in animal experiments
	DNase I	Degrades the NETs backbone and alleviates NETs-mediated pathological damage	Mouse APS model	Effective in animal experiments
Human experiment	Hydroxychloroquine (HCQ)	Inhibits complement activation, reduces aPL titers, and improves endothelial function	Clinical cohort studies, clinical observations	Routinely used in clinical practice for APS; recommended by guidelines
	Rituximab	Anti-CD20 monoclonal antibody; depletes B cells and suppresses autoantibody production	Case reports and small-sample clinical studies in severe APS	Clinically used for refractory APS
	Eculizumab	Complement C5 inhibitor; blocks the core step of complement activation	Case reports in CAPS	Clinically used for refractory CAPS
	Coenzyme Q10	Reduces peroxidase production and inhibits NETs formation	Small-sample clinical observation	Clinical exploratory stage

**Table 2:** Summary of drugs targeting NETs for obstetric treatment in APS.

Experimental type	Drug/intervention	Mechanism of action	Experimental model	Application status
Cell experiment	N-acetylcysteine (NAC)	Scavenges ROS and blocks the formation of NETs	*In vitro* trophoblast cells + APS-IgG injury model	Preclinical study
Animal experiment	PAD4 inhibitors (Cl-amidine, GSK484)	Inhibit PAD4 enzyme activity and reduce NETs release	Obstetric APS mouse model	Effective in animal experiments
	DNase I	Degrades the NETs backbone and alleviates local placental NETs-induced damage	Mouse obstetric APS model	Effective in animal experiments
Human experiment	Hydroxychloroquine (HCQ)	Inhibits complement activation, improves placental function, and reduces aPL titers	Obstetric APS clinical cohort, pregnancy outcome study	Routinely used in combination with anticoagulants for obstetric APS in clinical practice
	Coenzyme Q10	Reduces peroxidase production and inhibits NETs formation	Small-sample clinical observation of obstetric APS	Clinical exploratory stage

## Summary and outlook

NETs have been confirmed as a core effector link in APS thrombosis and obstetric complications. APLs trigger NETsosis via pathways like TLR4/NOX. The released DNA-protein scaffold simultaneously activates extrinsic TF, intrinsic FXII, and complement C5a. Its histones, NE, and MPO further damage endothelium, inhibit fibrinolysis, and aggregate platelets, forming a hypercoagulable microenvironment. Local placental NETs deposition leads to spiral artery remodeling failure through oxidative stress and trophoblast apoptosis, causing RFL, preeclampsia, and PTB. However, the quantitative relationship between antibody profile-NETs phenotype-clinical phenotype has not yet been established. Anti-NETs antibodies could potentially become a new class of clinical biomarkers, allowing more effective risk stratification and subphenotyping of aPL-positive individuals. More clinical trials are needed to evaluate the safety and efficacy of NETs-targeting therapeutic strategies, providing more precise treatment options for APS patients. Furthermore, research on NETs-based biomarkers holds promise for providing new tools for early diagnosis and disease monitoring in APS. With a deepening understanding of APS pathological mechanisms, treatments targeting NETs are expected to become a new breakthrough point in APS therapy, improving patient prognosis.
